# Spark plasma sintering of alumina nanopowders produced by electrical explosion of wires

**DOI:** 10.1186/s40064-015-1383-y

**Published:** 2015-10-06

**Authors:** Vladimir An, Alexey Khasanov, Charles de Izarra

**Affiliations:** Institute of High Technology Physics, National Research Tomsk Polytechnic University, 30 Lenin Ave., 634050 Tomsk, Russia; Groupe de Recherches sur l’Energétique des Milieux Ionisés (GREMI), 4 rue d’Issoudun, BP6744, 45067 Orléans Cedex 2, France

**Keywords:** Alumina nanopowders, Electrical explosion of wires, Spark plasma sintering

## Abstract

Alumina nanopowders produced by electrical explosion of wires were sintered using the spark plasma sintering technique. The results of XRD analysis show that the main phase in the compacted nanopowders is α-Al_2_O_3_. According to the SEM observations, the sintered alumina nanopowder consists of micron-sized faceted grains and nano-sized necked grains. The increase in sintering temperature resulted in a higher density of the sintered powders: from 78.44 to 98.21 % of theoretical density.

## Background

For ceramic technologies, it is important to obtain ultrafine microstructure that ensures improved physical and physic-mechanical properties: hardness, wear resistance, mechanical and optical properties. Over the last decade, spark plasma sintering has become a powerful technique to produce high quality ceramics including nanostructured ceramics (Bordia and Olevsky 2010; Angerer et al. 2006; Suárez et al. 2013; Monnier et al. 2015; Huang and Nayak 2014). Alumina has attracted great interest and is one of the most used materials in various applications. These materials display excellent properties: high strength, good chemical durability and excellent electrical insulating properties. They can be used as translucent ceramics (Wei 2005; Mao et al. 2008), thermal insulation (Xu et al. 2015), catalysts (Nartova et al. 2015; Gündüz and Dogu 2015), biomedical implant (Deville et al. 2003) etc.

In this work, spark plasma sintering (SPS) is considered as a promising fabrication way. The SPS technique has some advantages with respect to usual sintering methods: higher heating rates and local temperature gradients, particular local temperature distributions. The problem to be solved in the study is how the SPS parameters (pressure, temperature) are related to the product characteristics (size, morphology, porosity). One of the interesting methods for fabrication of alumina nanopowders is electrical explosion of wires (Yavorovskii 1996). The objective of this work was to study processes of densification during spark plasma sintering of alumina nanopowders produced by electrical explosion of wires (EEW) in the gaseous mixture of argon and oxygen at a pressure of 1.5 atm. The authors wanted also to explore polymorph transformations during spark plasma sintering of alumina nanopowders.

## Results and discussion

This research work consisted of four main stages: fabrication of alumina nanopowders by electrical explosion of aluminum wires, characterization of as-prepared nanopowders, spark plasma sintering of alumina nanopowders and their characterization. The specific feature of these experiments is the use of alumina nanopowders produced by electrical explosion of wires.

The method of electrical explosion of aluminum wires in the mixture of argon and oxygen was used for the preparation of alumina nanopowders. For this work aluminum wires having the diameter of 0.35 mm were employed. The wire (l = 65 mm) was input in the explosion chamber using a special feeding mechanism. The following parameters of electrical explosion were used: working gas pressure—1.5 atm, voltage—24 kV, capacity—2.3 µF, and inductance—0.72 µH. The BET analysis results showed that the specific surface area of as-prepared nanopowder was 20 m^2^/g.

**Fig. 1:**
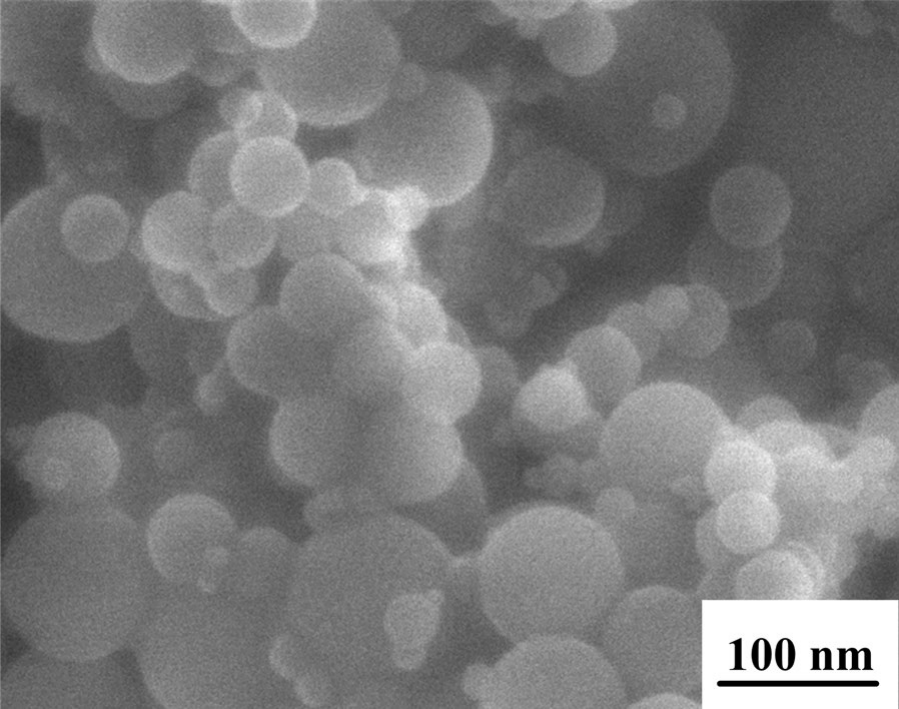
SEM micrograph of the alumina nanopowder produced by electrical explosion of aluminum wires in the mixture of argon and oxygen

The product of this process is alumina nanopowder. Figure 1 illustrates the alumina nanopowder prepared using the EEW technique in the mixture of argon and oxygen. It is well known that metal nanopowders produced by EEW can reveal certain deficiency of crystalline structure. The shape of prepared particles seems to be rather spherical. However some of these particles can have a faceted surface. The faceted particle surface is explained by structural defects in electroexplosive nanopowders and features of surface aluminum oxide shell.

**Fig. 2:**
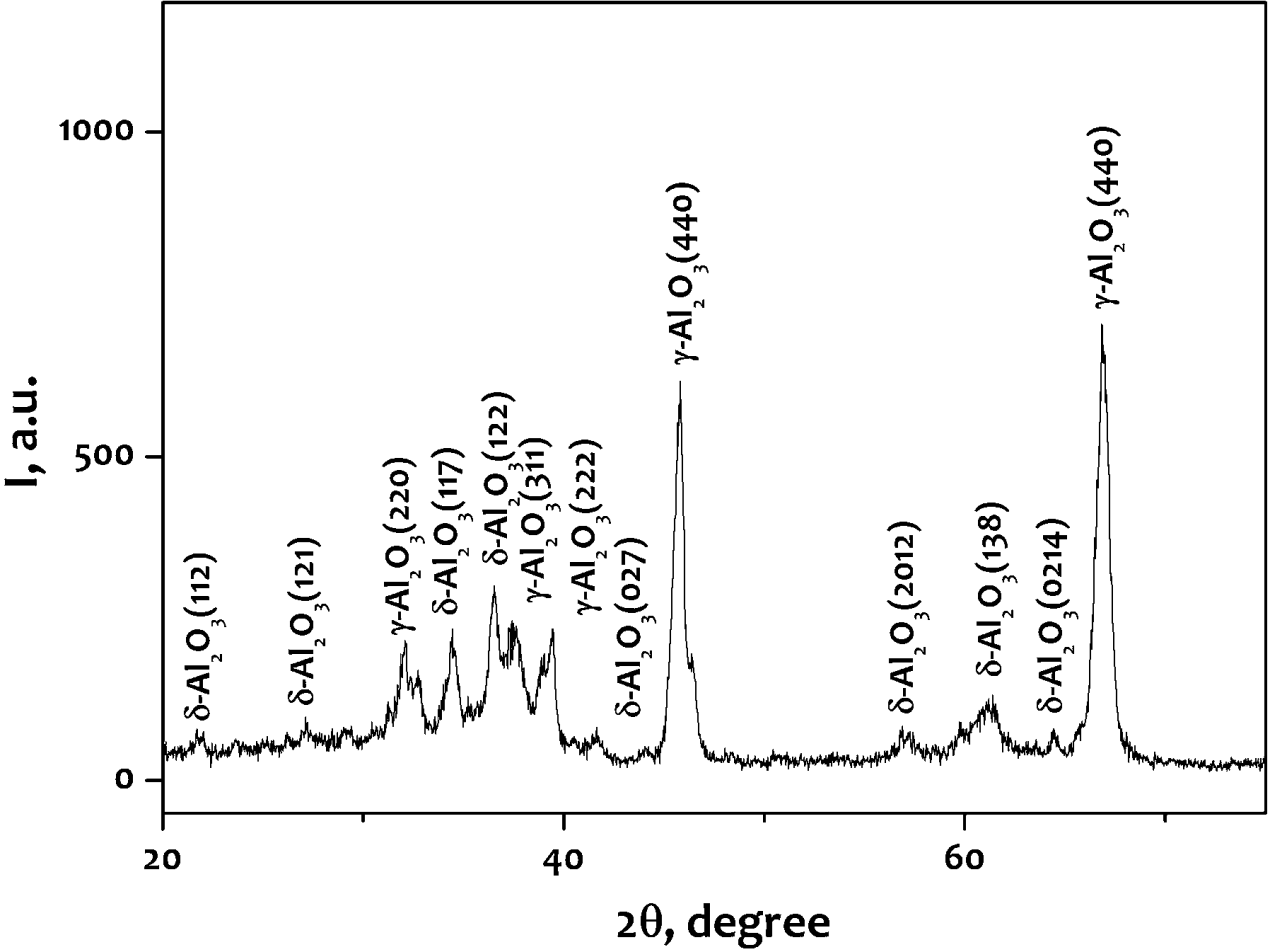
X-ray pattern of the alumina nanopowder produced by electrical explosion of wires

XRD analysis was carried out in order to characterize the phase and crystal structure of the alumina nanopowder samples produced by electrical explosion of wires. The X-ray pattern of the sample (Fig. 2) was analyzed to detect different polymorph alumina structures in the as-prepared powder. According to the data of X-ray analysis, the main phases in the products are γ-Al_2_O_3_ and δ-Al_2_O_3_. According to the intensity of peaks, the dominant phase is γ-Al_2_O_3_.

**Fig. 3:**
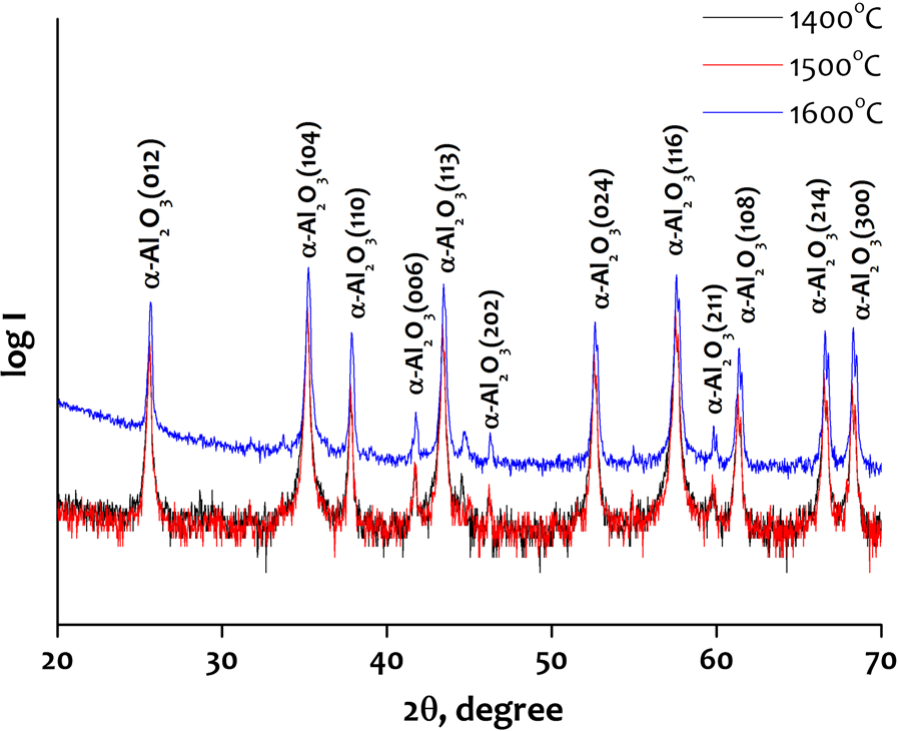
SEM micrograph of the alumina nanopowder sintered by SPS at 1400 °C using a pressure of 40 MPa

**Fig. 4:**
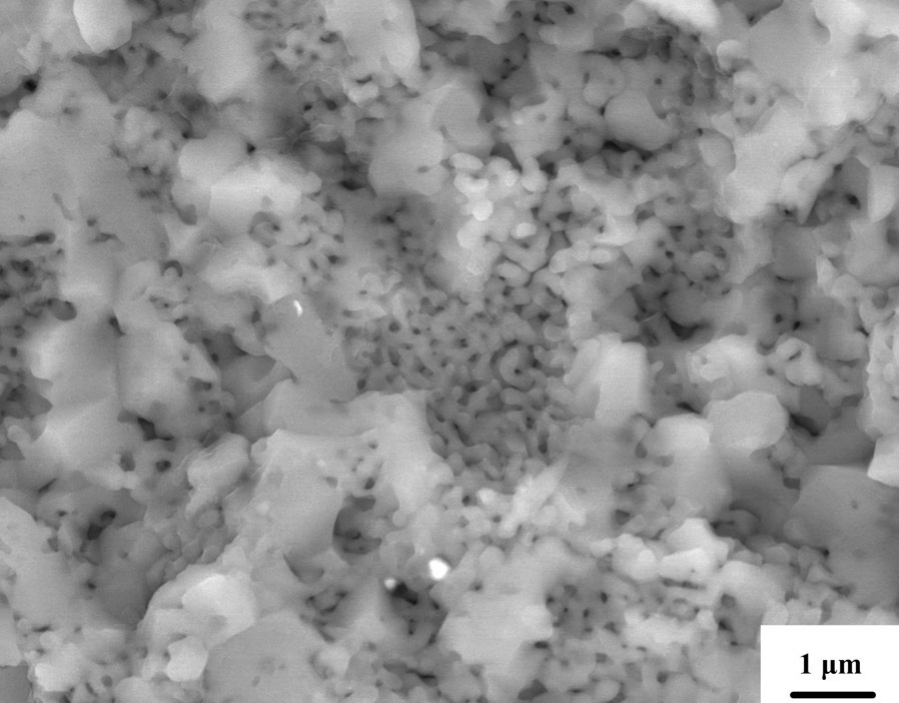
X-ray patterns of the alumina nanopowder sintered by SPS at 1400 °C

The alumina sintering processes reveal a dual nature. On one hand, the SEM micrograph in Fig. 3 shows that the image of alumina sintered at a temperature of 1400 °C and a pressure of 40 MPa. This micrograph shows that the sintered body consists of micron-sized faceted grains and nanosized necked grains. SPS relates to grain-boundary diffusion and migration processes connected to the electric field impact. It can be assumed that the migration processes can be related to the employment of higher temperatures and pressures. Electric field can also impact significantly on the growth of alumina nanopowder grains. The X-ray analysis shows that the γ-Al_2_O_3_ and δ-Al_2_O_3_ phases disappeared in the final products of alumina nanopowder sintering. The only phase found in the sintered alumina nanopowder was α-Al_2_O_3_ (Fig. 4). This fact is explained by normal polymorph transformations in alumina when heating it up to temperatures higher than 1400 °C. The presence of γ-Al_2_O_3_ and δ-Al_2_O_3_ phases in electroexplosive alumina nanopowders is related to special fabrication conditions which provide strong non-equilibrium allowing stabilization of low-temperature modifications in the final products. Spark plasma sintering provokes relaxation processes leading to transformation into more stable high-temperature alumina phases.

Table 1 shows the densification parameters of alumina nanopowder sintered at different temperatures by the SPS technique. According to the table, the apparent density of the sintered pellets increases with an increase in the sintering temperature. Finally, the density reaches the value quite close to the theoretical density—98.21 %. It witnesses the rather good effectiveness of the SPS technique even for the comparatively low sintering pressure of 40 MPa. To reach the highest density (more than 99.5 %), further experiments with a higher pressure are supposed to be conducted using dyes allowing higher pressures.

**Table 1:** Densification parameters of alumina nanopowder sintered at different temperatures by the SPS technique

Temperature, °C	1400	1500	1600
Absolute density ρ, g/cm^3^	3.11	3.70	3.90
Relative density ρ, %	78.44	93.32	98.21

## Conclusions

Alumina nanopowders were sintered using the spark plasma sintering technique. The samples were sintered at a pressure of 40 MPa and a temperature of 1400, 1500 and 1600 °C. The SPS processes support essential the densification processes. The increase in the sintering temperature leads to a higher density of sintered alumina nanopowder: the relative density increased from 78.44 to 98.21 %. Sintering resulted in polymorph transformation of the initial alumina nanopowder (a mixture of γ-Al_2_O_3_ and δ-Al_2_O_3_) into pure α-Al_2_O_3_.

## Experimental

**Fig. 5:**
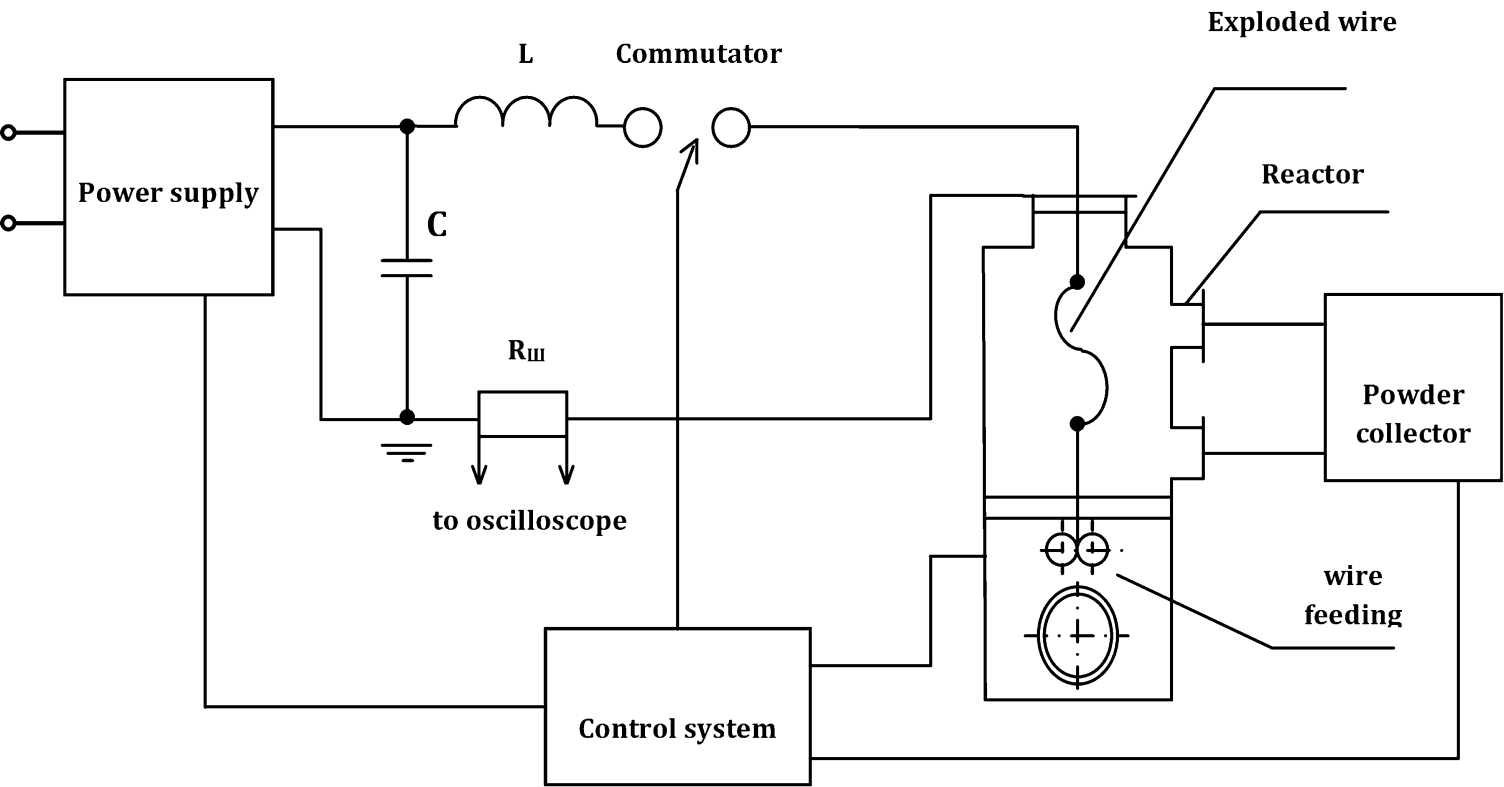
Scheme of the experimental setup for fabrication of alumina nanopowders

Alumina nanopowders (NP) were produced by electrical explosion of aluminum wires in the mixture of argon and oxygen (Yavorovskii 1996). The EEW is connected to a high current density (j > 10^10^ A/m^2^) and a high metal heating rate (~10^10^ K/s) characterizing the process up to high temperatures (T > 10^4^ K). The EEW method allows manufacturing a large variety of metals and compounds, including alumina nanoparticles used in this study. Electrical explosion of wires is carried out in the setup the principal scheme of which is given in Fig. 5. The EEW technological module consists of high voltage input, commutator, reactor, wire feeding mechanism, working gas inlet system, control system, gas circulation device and powder collector.

Nanopowders produced by EEW reveal higher chemical reactivity (An et al. 2010), unusual physicochemical properties (An et al. 2011), can be used for fabrication of nanostructured compounds (Bozheyev et al. 2014) and for other applications (An and Irtegov 2014). In the present work, alumina nanopowders produced by electrical explosion of alumina wires were used in the experiments of spark plasma sintering.

**Fig. 6:**
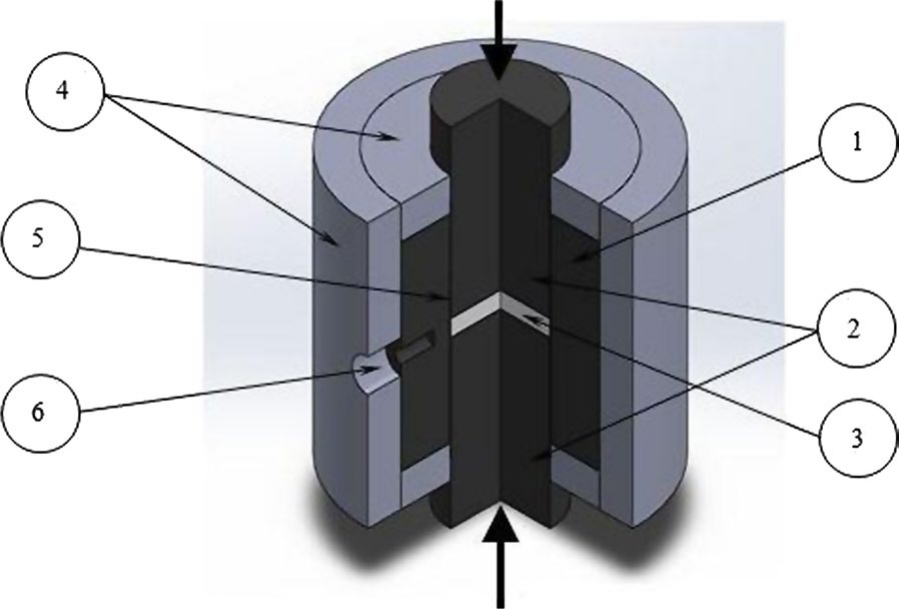
The SPS graphite die with a sample: *1* die, *2* punches, *3* sample, *4* graphite felt, *5* graphite washer, *6* pyrometer hole

The SPS graphite die with the sample configuration are shown in Fig. 6. The temperature was measured by an optical pyrometer focused on the surface of the graphite die and automatically regulated from 580 °C up to the final sintering temperatures of 1400, 1500, 1600 °C.

A Shimadzu 7000S X-ray diffractometer with CuK_α_ radiation (λ = 1.54 Å) was used for X-ray phase and structural analysis. A JSM-7500FA scanning electron microscope (JEOL, Japan) was used to observe the morphology and structure of the synthesized nanopowders.
